# Challenges and opportunities for large-scale electrophysiology with Neuropixels probes

**DOI:** 10.1016/j.conb.2018.01.009

**Published:** 2018-06

**Authors:** Nicholas A Steinmetz, Christof Koch, Kenneth D Harris, Matteo Carandini

**Affiliations:** 1 University College London, London, UK; 2 Allen Institute for Brain Science, Seattle, WA, United States

## Abstract

Electrophysiological methods are the gold standard in neuroscience because they reveal the activity of individual neurons at high temporal resolution and in arbitrary brain locations. Microelectrode arrays based on complementary metal-oxide semiconductor (CMOS) technology, such as Neuropixels probes, look set to transform these methods. Neuropixels probes provide ∼1000 recording sites on an extremely narrow shank, with on-board amplification, digitization, and multiplexing. They deliver low-noise recordings from hundreds of neurons, providing a step change in the type of data available to neuroscientists. Here we discuss the opportunities afforded by these probes for large-scale electrophysiology, the challenges associated with data processing and anatomical localization, and avenues for further improvements of the technology.

**Current Opinion in Neurobiology** 2018, **50**:92–100

This review comes from a themed issue on **Neurotechnologies**

Edited by **Polina Anikeeva** and **Liqun Luo**

For a complete overview see the Issue and the Editorial

Available online 13th February 2018

**https://doi.org/10.1016/j.conb.2018.01.009**

0959-4388/© 2018 The Authors. Published by Elsevier Ltd. This is an open access article under the CC BY license (http://creativecommons.org/licenses/by/4.0/).

Technologies to record the spikes of individual neurons *in vivo* have shown an accelerating improvement over the last five decades, with the number of recording sites per electrode shank growing from 1 to 1000 (Figure 1a). Recordings from individual neurons *in vivo* began in earnest with insulated metal microelectrodes such as those made of indium [1] and tungsten [2], which were robust and practical to construct. These electrodes typically record from one neuron at a time: when their small, high-impedance tip is placed very close to a neuron, they isolate its activity extremely well by making its spikes larger than those of its neighbors.

**Figure 1:**
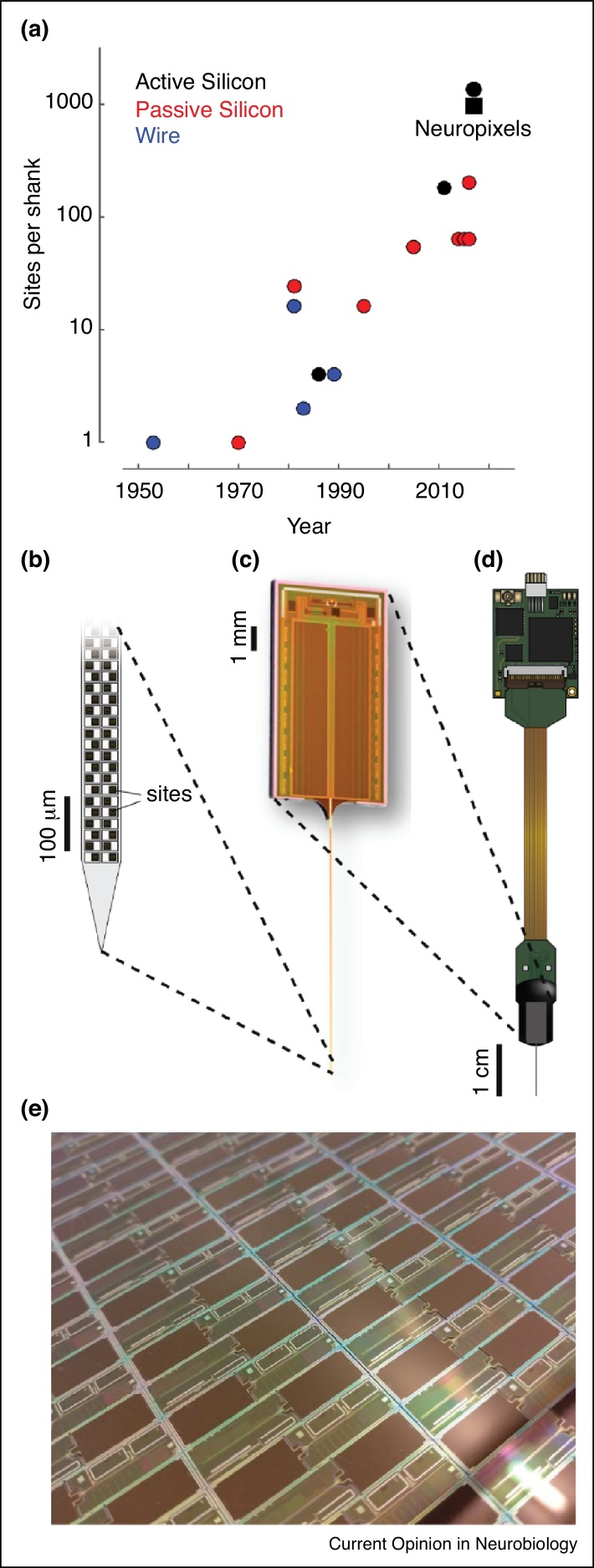
Growth of electrode technology leading to Neuropixels. **(a)** Sites per shank over time for a selection of devices. Devices without successful *in vivo* demonstrations are excluded. Blue, devices made from wires, refs [1, 3, 5, 7]. Red, passive silicon, refs [11, 12, 13, 14, 15, 16, 17, 18, 19]. Black, active silicon, refs [15, 20••, 21•, 22] (square icon indicates Neuropixels). **(b)**–**(d)** The Neuropixels probe. (b) Schematic of tip, showing sites arranged in dense checkerboard pattern. (c) The printed CMOS element, including the shank as well as circuitry implementing amplification, multiplexing, and digitization. (d) The packaged device, with flex cable and headstage for interfacing and further multiplexing. **(e)** Neuropixels probes on CMOS wafer. Panels (b and d) are reprinted with permission from Ref. [20^••^].

A breakthrough in population recording came with the introduction of the tetrode [3, 4, 5, 6]: a bundle of larger, low-impedance metal microwires twisted together so their recording sites are closely spaced. The larger size and lower impedance of these sites allows up to ∼20 neurons to be recorded simultaneously, and the spikes of these different neurons can be discriminated through differences in amplitude and waveform across sites. However, adding recording sites to these bundles requires adding microwires, which makes the device more invasive. Indeed, although such devices can have 16 or more recording sites per bundle [7, 8], they are generally too wide for most applications *in vivo*. However, if the displacement of tissue and consequent damage to neurons and glia is deemed acceptable, a possible strategy is to insert dozens or hundreds of microwires, thus recording from large populations of neurons [9, 10].

Increasing channel count while maintaining a reasonable device size required adopting silicon microfabrication techniques. These techniques led to silicon probes that featured micron-scale recording sites and insulated recording lines [11]. The critical constraint for such probes is the number of recording lines (i.e. independent electrical connections) that can pass along the probe shank, which limits the number of channels that can be simultaneously recorded. Through miniaturization, it became possible to fit more lines, and therefore more sites, on a single shank while maintaining a reasonable width (≥16 sites; [12, 14]). A notable alternative design, the Utah array, made use of silicon fabrication techniques to produce a many-shank ‘bed of nails’ array, one that has become the device of choice for human-implanted brain-computer interfaces [23, 24, 25].

However, the costs associated with state-of-the-art fabrication equipment and the labor-intensive manufacturing process limited development of silicon devices with still higher numbers of channels. This difficulty was overcome by using nanofabrication processes such as electron-beam lithography, which delivered devices with up to 200 recording sites on a thin shank [17, 18]. Such devices face a different limitation: connecting the probe to external amplifiers requires vast numbers of interconnect cables, a challenge that precludes their use as a chronically implanted device in small animals such as rodents.

To reduce the number of interconnects, it is necessary to use multiplexing, so that signals from multiple recording sites travel along the same cable. This can be achieved by making probes that are active, i.e. which receive power, and incorporate the necessary electronics. Using this approach, probes were constructed that contained electronics not only for multiplexing but also for operations such as digitization, generation of electrical stimuli, and spike detection [15, 26, 27, 28, 29].

These and other advances were combined to obtain Neuropixels probes, developed by T. Harris and colleagues [20^••^], which allow larger numbers of channels to be recorded than was previously possible, at low cost (forecast to be on the order of €1000/probe). These probes are based on the complementary metal-oxide-semiconductor (CMOS) technology that is used for constructing silicon integrated circuits. This semiconductor technology dramatically reduces wire width (to 130 nm in the probes) and allows the probe to contain all the active circuits needed for amplification, digitization, and multiplexing [20••, 30]. The result is a device that has 960 recording sites (384 configurable recording channels) on a 70 × 20 μm shank, weighs only ∼0.3 g, and produces data that are already amplified and digitized (Figure 1b–d). Perhaps as importantly, the high-throughput, scalable CMOS fabrication techniques allow the device to be cheaply made in volumes suitable for wide distribution among the community (Figure 1e). A related probe, called Neuroseeker [21^•^], used similar technology with an even higher electrode count but with substantially higher noise levels (Table 1) and with issues of light sensitivity, as even ambient light could create artifacts.

**Table 1:** Summary of key parameters of technologies for large-scale electrophysiology

Name	Technology	Site material	Shank cross-section (μm)	Shank length (mm)	Shanks per probe	Sites per shank	Recordable sites	Density (sites/mm)	Volume per channel (10^3^ × μm^3^)	Recording span (mm)	Noise level (μV)	Probe weight (g)	Headstage weight (g)	Digital output
Neuropixels [20^••^]	Active Si	TiN	70 × 20	10.0	1	960	384	100	14	3.8	5.5	0.3	1.1	Y
Neuroseeker [21^•^]	Active Si	TiN	100 × 50	8.0	1	1356	1356	170	30	7.8	31.0	0.1	1.3	Y
Neurotech Alliance [18]	Passive Si	Gold	24–100 × 21	5.0	16	64	1024	100	42	0.6	4.8	6.8	4.8	N
E-beam [17]	Passive Si	PEDOT-plated Gold	40–120 × 15	7.5	5	204	1020	154	9	1.3	4–8	25.1	n/a	N
Cambridge Neurotech [31]	Passive Si	Conductive polymer	30–78 × 15	8.0	1	64	64	50	20	1.3	?	0.5	1.3	N
Silicon microprobes [19, 32]	Passive Si	Electroplated Gold	86 × 23	7.0	4	64	256	61	46	1.1	3.0	1.3	2.6	N
Utah array [23]	Passive Si	IrOx	23–106 (diam.)	1.0	100	1	100	n/a	3268	n/a	?	0.02	1.0	N
Neuronexus [16]	Passive Si	Ir	20–96 × 15	5.0	8	32	256	20	44	1.6	?	0.3	4.7	N
Wire tetrode [33] (with flexDrive, [34])	Wire	Gold	∼40 (diam.)	5.0	16	4	64	n/a	471	n/a	3.0	2	1.4	N
Microwire bundles [10]	Wire	Steel	50 (diam.)	20.0	128	1	128	n/a	2950	n/a	20.0	?	?	N

Where different models of probe are available, the following were used: Neurotech Alliance, G1-P07; Cambridge Neurotech, H3; Neuronexus, Buzsaki256. Shank cross-sections are rectangular with given dimensions except where ‘diam.’ (diameter) is specified, for which the cross-section is approximately circular. A range of numbers indicates that the probe tapers from a thick to a thinner cross-section at the tip. Recordable sites are the number of total channels simultaneously recordable with one probe (in some cases including multiple shanks), given appropriate recording hardware. Density refers to the number of sites per millimeter along a single shank. Volume per channel indicates the total displaced volume per channel in the brain, for an insertion depth of 1.5 mm (except Utah array, for which insertion depth is the maximal 1.2 mm), calculated from given dimensions. For scale, a cell body with diameter 10 µm occupies about 0.5 × 10^3^ μm^3^. Recording span indicates the distance that can be recorded on a single shank at the specified density. Site material abbreviations: TiN, titanium nitride; IrOx, iridium oxide; Ir, iridium; Pt/Ir, platinum/iridium. Noise levels are root-mean-square, measured end-to-end, and only included where explicitly reported in the referenced publication. For Neuroseeker, note that a lower noise (12.4 μV RMS) is available when choosing to record from only half of the stated number of channels. Headstage weight for Cambridge Neurotech assumes Intan RHD2132. Digital output indicates the format of data produced by the probe, i.e. whether the data has already been amplified and digitized on probe (Y) or whether the output is the raw voltage such that further hardware is required to acquire data (N).

A selection of key characteristics of the technologies currently used for large-scale electrophysiology, including Neuropixels, is provided in Table 1. In these characteristics, Neuropixels probes are comparable or superior to other devices. Moreover, Neuropixels probes have other desirable characteristics, such as path to commercialization, lower system cost (no additional amplifiers required), highly consistent site-to-site impedance, precisely straight shanks (<50 μm deviation over 10 mm), small and thin base size (allowing multiple probes closely adjacent), and elimination of cable-related motion artifacts (due to on-probe digitization). Given these attractive characteristics, in the following we concentrate on Neuropixels probes, and discuss the opportunities and challenges that they provide.

## Opportunities: unprecedented data sets

Neuropixels probes present new opportunities for neuroscience. By sampling signals densely, they isolate single neuron activity better than previous technologies, often detecting spikes from individual neurons on more than a dozen sites [20^••^]. Moreover, since there are no gaps in their dense coverage of a ∼4 mm span of recording sites, neurons will be reliably recorded at every point along the trajectory. The selection of sites is configurable, so an even longer recording span is possible: halving the density doubles the length of the recording array to ∼7.7 mm.

**Figure 2:**
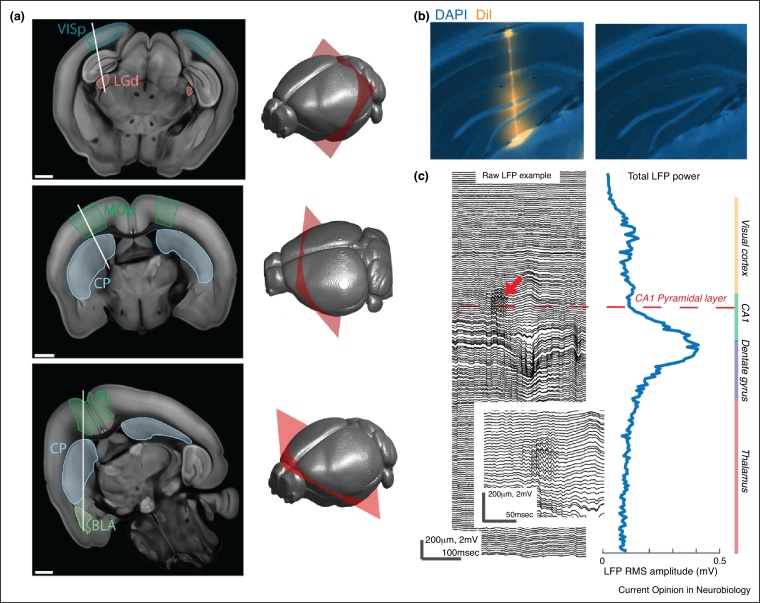
Neuropixels penetrations through the brain. **(a)** Example recording vectors that can be achieved with single Neuropixels probes. Left, Oblique sections through a reference brain atlas with hypothetical probe tracks illustrated in white. Right, Locations of the sections (red) shown at left. VISp, primary visual cortex; LGd, dorsal lateral geniculate nucleus; MOp, primary motor cortex; CP, caudoputamen; PL, prelimbic cortex; BLA, basolateral amygdala. All scale bars 1 mm. The Allen Institute Common Coordinate Framework reference atlas was used to generate these images. **(b)** Histological reconstruction of an actual probe track, showing DAPI stain (blue) without (top) and with (bottom) overlay of the fluorescent indicator DiI (orange) used to coat the probe. Due to small shank dimensions (70 × 20 μm), Neuropixels probes must be localized with a dye or with functional signatures. **(c)** Example LFP recording and features that can be used to localize probe sites. Left, sample of raw LFP signal from a subset of channels with sharp-wave ripple indicated (red arrow) and zoomed-in (insert). Right, total LFP power averaged over the recording, showing peak in dentate gyrus. Panel c is reprinted with permission from Ref. [20^••^].

In a small brain such as that of the mouse, this coverage enables dense sampling of multiple brain structures simultaneously (Figure 2a). For instance, a Neuropixels probe can simultaneously record from all layers of neocortex and hippocampus, from all layers of superior colliculus along with periaqueductal gray, or from a set of adjacent functionally related areas such as cingulate, prelimbic, and infralimbic cortex. A single probe can thus record both input and output structures of a processing stream, such as the lateral geniculate nucleus and primary visual cortex (Figure 2a, top), or primary motor cortex and striatum (Figure 2a, middle). If the longer recording span is chosen, a single probe can record even more distant structures, such as prefrontal cortex and basolateral amygdala (Figure 2a, bottom).

Because the probes include the entire recording system, they require minimal cabling — a single data line from each probe. Multiple probes can thus be used simultaneously in close apposition, allowing them to reach any combination of brain regions. For instance, eight probes can be inserted simultaneously into the brain of a head-fixed mouse, enabling the recording of more than 3000 sites in an awake mammal, approximately an order of magnitude greater than with any previous technology [35]. The scale of these recordings reveals the dynamics of neural activity across large swaths of brain at millisecond temporal resolution and single neuron spatial resolution. The size of the recorded populations may enable the study of neural computation at a single-trial level during behavioral tasks, and may allow effective study of connected pairs of neurons, which typically represent only a small fraction of recorded pairs (<1%; [36, 37]). Finally, the probes’ are small enough for chronic implantations even in small mammals such as mice, and minimizes geometric constraints for experiments such as simultaneous electrophysiology and calcium imaging.

This new scale of recording promises a new era in neuroscience, in which we are no longer limited for technical reasons to studying the activity of few neurons in only one or a few brain regions. Instead, we can now study simultaneously a large fraction of the neuronal populations relevant for behavior, revealing the dynamics of the recurrently connected circuits and systems that underlie behavior.

## Challenges: data processing

Neuropixels probes produce large amounts of data, which need to be processed to assign spikes to individual neurons (spike sorting, [38]). The data acquisition rate (∼1 GB/min for 384 channels at 30 kHz) is more similar to that seen in imaging than in electrophysiology, providing some challenges for data storage. However, the main computational challenge is that of performing spike sorting. To meet this challenge, new algorithms have been developed, which take advantage of inexpensive computational resources such as GPUs and incorporate novel algorithmic steps [39•, 40, 41, 42, 43, 44].

Despite these algorithmic advances, no spike sorting algorithms are yet truly automatic, requiring manual supervision to improve results. One reason for this is the problem of electrode drift, the movement of the brain relative to the probe. Algorithmic approaches can be taken to join groups of spikes whose shapes have shifted over time [45, 46], but Neuropixels probes enable a different solution to this problem: registration of the raw data across time to undo the effects of drift. This signal registration is analogous to image registration in imaging experiments, where images are corrected for brain movement before further processing. Because Neuropixels probes sample densely along their trajectory, motion of the brain relative to the probe is visible as spikes simply shift up or down from one site to the next, which could be automatically corrected with registration methods.

However, the biggest impediment to developing fully-automatic spike sorting algorithms is the difficulty of ground truth validation. No algorithm can be trusted blindly without quantitative evidence that it has low error rates, and the development of metrics to assess these error rates is an area of active research [41, 47]. Ultimately, however, such metrics require ground truth. One way to obtain this ground truth is through detailed simulations of spiking neurons, which produce synthetic voltage traces with predetermined spike times [48], but the simulations will only be as realistic as the designer knows how to make them. Another approach is to construct ‘hybrid ground truth’ datasets, where real spike waveforms recorded on one part of the probe are de-noised, subtracted, and then added back at predetermined times to a different part of the probe [39^•^]. This approach preserves the true spike waveforms and spike-to-spike variability, but its success depends on the way the ‘donor’ spikes were sorted in the first place. Moreover, the inserted spikes may no longer occur at the same time or same spatial position as extraneous, confounding electrophysiological events that may have affected the original spikes.

The best form of ground truth, then, is actual simultaneous recording of spikes with another method such as a juxtacellular electrode [49•, 50]. This approach too has its limitations. First, the conditions under which the ground truth is recorded (e.g. anesthesia) may not match the desired experimental conditions. Second, and most importantly, this approach is technically difficult, and can thus produce only small datasets. In the future, imaging of membrane-localized voltage sensors [51] or methods to elicit single spikes from individual neurons [52] may provide new ways to collect ground truth with higher throughput.

Given this context, careful manual curation of the results of spike sorting algorithms is still critical, and public, open-source software packages have been developed to improve the efficiency of this process [53]. Even after manual curation, however, it is important to keep in mind the errors that may result from erroneously assigning the spikes of two neurons to a single cell, from missing the occurrence of some spikes from a neuron, or from having error rates that co-vary with brain motion. When these sources of error are plausible, careful steps must be taken to ensure that they do not influence the scientific findings. For instance, one may plot the size of a putative scientific effect against the quality of sorted neurons, and determine whether or not the effect asymptotes at high quality [54].

## Challenges: probe localization

Targeting a probe to a desired brain structure and subsequently localizing the recording sites is a challenge for any electrophysiology experiment. With Neuropixels probes, targeting is easier: it is difficult to miss the desired brain location along the insertion vector, given that all sites over a 4 mm span along that vector will be recorded when using the densest site configuration. Localization, however, is a challenge. Neuropixels probes cannot produce electrolytic lesions, and their relatively small cross-section may not leave visible tracks through DAPI- or Nissl-stained tissue (Figure 2b). Fortunately, the probes are compatible with another classic localization technique, the application of a fluorescent dye to the probe before recording followed by subsequent slicing and fluorescence imaging. The dyes that are typically used are lipophilic dyes (such as DiI). For use in tissue-cleared brains, one could adopt fluorescent dyes modified to also adhere to proteins [55, 56].

To determine the brain regions in which neurons were recorded, one could then proceed as usual by manually identifying each structure along the recording track. However, this problem may be solved another way using recently developed 3D atlases of the mouse brain (Allen Institute's “Common Coordinate Framework” or the Waxholm Space, [57]). After registering histological images to the atlas (e.g. [58]) or simply by identifying manually the 3D coordinates of the observed fluorescent dye, the labels of the areas may be read out from the atlas and compared to the data from the recording.

Neuropixels probes may also offer the opportunity to systematically take advantage of the electrophysiological signatures of brain regions and layers that have long been known ‘by ear’ to electrophysiologists (Figure 2c). With a database of Neuropixels recordings registered to an anatomical atlas, an ‘electrophysiological brain atlas’ could be constructed to allow automated identification of the recording sites based on these signatures alone, and constrained by their relative locations on the probe.

## Outlook: future probe technologies

Neuropixels probes are manufactured with CMOS technologies, so they are inexpensive to produce in large volumes and open to significant further improvement. As circuit fabrication technologies improve, the device size will shrink. For instance, a version of the probe with a significantly smaller base area may be possible, and would increase the utility of Neuropixels probes for chronic implantations in small animals such as mice.

When light is delivered extrinsically, Neuropixels probes are compatible with optogenetic experiments involving activation of local circuitry or opto-tagging, i.e. the identification of neurons on the basis of their expression of genetically-encoded light-activatable channels [20^••^]. However, existing technologies also allow the integration of light sources with neural probes [59••, 60] for optogenetic manipulations. A probe combining these technologies with the dense and extensive recording capabilities of Neuropixels probes would be ideal for such experiments. On-probe light-emitting technology, in the future, could even be combined with light detection on the same probe to achieve functional imaging deep in the brain [61].

Certain use cases are not ideally served by Neuropixels probes. First, chronic implants in primates or other large animals require probes that have either flexible shafts or that ‘float’ on the brain to minimize tissue-probe movement, which can damage either or both. Research into materials and designs for such applications is ongoing [62, 63] but largely parallel to the development of CMOS probes like Neuropixels due to the incompatibility of most of those materials with the high-density CMOS manufacturing methods. Moreover, though chronic implants of Neuropixels probes in rats and mice were demonstrated to last up to five months [20^••^], additional studies are required to assess suitability for longer implants. Implants of a year or more may require different designs or materials for increased long-term biocompatibility. Second, a probe with a single long shank is not ideally suited to certain recording geometries. For example, in an experiment whose goal was to record the largest possible number of neurons from a thin, horizontally-elongated structure such as dorsal CA1 in rodent hippocampus, multi-tetrode systems [64, 65] or silicon probes with custom geometries may still be preferable. A multi-shank version of the Neuropixels probe would better meet these recording requirements. Third, Neuropixels probes are incapable of electrical microstimulation, a technique classically employed both for electrode localization and for probing the role of neural circuits in perception and cognition [66, 67, 68].

To record from greater numbers of sites simultaneously, other projects have taken advantage of the capabilities of CMOS electronics to develop probes that rapidly switch between recording sites [21•, 69]. Though these probes exhibit problematic noise levels and light sensitivity, future developments may nevertheless make this approach workable. Another inventive use of CMOS technology is the repurposing of large arrays of tiny amplifiers, such as the pixels on a CMOS camera sensor, to work as recording channels for neural signals by coupling them with large bundles of microwires, thus enabling massive scaling of the number of microwires that could be simultaneously recorded [70]. Finally, even more creative solutions may ultimately push channel counts still higher. For instance, if autonomous, wirelessly-transmitting single-channel recording systems could be made small enough (tens of microns), we might be able to sprinkle them like dust throughout the brain [71, 72, 73].

Thus electrophysiology enters an exciting new era, as datasets explode and our capability to measure global brain dynamics at fine spatial and temporal scales reaches new heights. Making sense of the resulting data explosion will be a major challenge, but that is a good problem to have.

## Conflict of interest statement

Nothing declared.

## References and recommended reading

Papers of particular interest, published within the period of review, have been highlighted as:

• of special interest
•• of outstanding interest
